# Bonobos Respond to Distress in Others: Consolation across the Age Spectrum

**DOI:** 10.1371/journal.pone.0055206

**Published:** 2013-01-30

**Authors:** Zanna Clay, Frans B. M. de Waal

**Affiliations:** Living Links, Yerkes National Primate Research Center, Emory University, Atlanta, Georgia, United States of America; Université de Strasbourg, France

## Abstract

How animals respond to conflict provides key insights into the evolution of socio-cognitive and emotional capacities. Evidence from apes has shown that, after social conflicts, bystanders approach victims of aggression to offer stress-alleviating contact behavior, a phenomenon known as *consolation*. This other-orientated behavior depends on sensitivity to the other's emotional state, whereby the consoler acts to ameliorate the other's situation. We examined post-conflict interactions in bonobos (*Pan paniscus*) to identify the determinants of consolation and reconciliation. Thirty-six semi-free bonobos of all ages were observed at the Lola ya Bonobo Sanctuary, DR Congo, using standardized Post-conflict/Matched Control methods. Across age and sex classes, bonobos consoled victims and reconciled after conflicts using a suite of affiliative and socio-sexual behaviors including embracing, touching, and mounting. Juveniles were more likely to console than adults, challenging the assumption that comfort-giving rests on advanced cognitive mechanisms that emerge only with age. Mother-reared individuals were more likely to console than orphans, highlighting the role of rearing in emotional development. Consistent with previous studies, bystanders were more likely to console relatives or closely bonded partners. Effects of kinship, affiliation and rearing were similarly indicated in patterns of reconciliation. Nearby bystanders were significantly more likely to contact victims than more distal ones, and consolation was more likely in non-food contexts than during feeding. The results did not provide convincing evidence that bystander contacts served for self-protection or as substitutes for reconciliation. Overall, results indicate that a suite of social, developmental and contextual factors underlie consolation and reconciliation in bonobos and that a sensitivity to the emotions of others and the ability to provide appropriate consolatory behaviors emerges early in development.

## Introduction

Understanding how animals respond to social conflict provides key insights into the dynamics of animal social relationships and underlying socio-emotional and cognitive processes, such as perspective-taking, empathy, and emotion regulation [1]. After aggressive conflicts, uninvolved bystanders in some species spontaneously approach an opponent to offer affiliation. Generally, the target of this contact is the victim, although bystanders may also approach the aggressor [2]–[3]. This form of other-directed behavior has aroused considerable debate in regards to both the function and the underlying mechanisms, in particular whether or not it may be driven by empathic processes, as opposed to other forms of emotional responding.

In some primates, offering affiliative contact to the victim is thought to function as a form of bystander-mediated reconciliation, notably if the bystander has a close relationship with the aggressor [4]–[5]. In other cases, providing affiliation may function as self-protection, whereby the affiliation serves as appeasement to prevent the bystander from becoming a victim of re-directed aggression ([6]–[7], but see [8]). In a few species, spontaneously receiving affiliative contact appears to reduce the victim's distress following the conflict. This phenomenon, known as *consolation* [9], is rare across the animal kingdom, so far having been demonstrated only in apes (*Pan troglodytes*) [2], [9]–[13]; (*P. paniscus*) [14]; *(Gorilla gorilla)* [15]–[16], as well as a few other animals known for their advanced social cognitive skills, such as corvids [17]–[18], canids, [19]–[20] and elephants [21]. Consolation is distinct from affiliative contact sought out by the victim in that the bystander actively offers reassurance after a conflict in which they played no role. De Waal & Aureli [22] were the first to propose that consolation may set apes apart from monkeys, since monkeys do not seem to show such behavior [23].

While the underlying mechanisms are still under debate, to spontaneously provide consolation is thought to require some level of other-awareness or emotional perspective-taking, which allows the bystander to both recognize the emotional state of the victim and to provide the appropriate response to reduce distress. Being able to experience another individual's emotions, while separating them from one's own, is considered a more cognitively demanding form of empathy, known as *sympathetic concern* [24]–[25]. In human development, for example, children from around age two increasingly exhibit cognitive, emotional and behavioral signs of concern for distressed others and appear to comprehend anothers' difficulties and act upon this by providing comfort and assistance [25]–[26].

While two years appears to be a key developmental milestone for empathy, prosocial behavior and related skills in separating the self from the other [25]–[31], recent evidence has indicated that forms of affective and cognitive empathy towards others in distress are already present before the second year [27]. Moreover, challenging the assumptions that young infants respond to others emotions invariably with personal distress, rather than sympathetic concern, it was shown that reactions of personal distress towards other's distress were actually rare in 8–16 month old infants [27]. Overall, the literature suggests that while more complex forms of cognitive empathy emerge in conjunction with developing cognitive skills, the foundations for other-orientated empathetic responding are already present in human infants from an early age. In addition, studies have also revealed that disruptions in development, brought on by infant neglect/deprivation or abuse, negatively affect the development of empathic behavior, attachment, and emotion regulation [32]. Currently, we know little about the development of emotional processing and prosociality in non-human primates or the role of rearing in consolatory behavior, a deficit that the current study seeks to address.

Parallels between the sympathetic concern of children and post-conflict consolation by apes concern both the context of the response and its morphology, since chimpanzees use similar affiliative behaviors (e.g. touching, embracing, kissing) as children do [11], [25]–[26]. Considering the close phylogenetic relationship between great apes and humans, a parsimonious assumption about such similarities is that the underlying psychological mechanisms are also similar [33]. As with other expressions of empathy, sympathetic concern is generally predicted by social closeness, familiarity and similarity between partners [24]. Consistent with this pattern, consolation in chimpanzees and other animals is promoted by social closeness of the bystander to the recipient in terms of kinship or affiliative bonds ([12]–[13], but see [2]). While patterns of chimpanzee consolation are consistent with empathy-based explanations used in the human developmental literature, the underlying mechanisms nevertheless remain hard to elucidate and alternative mechanisms, such as associative learning, should be considered as well. Moreover, whereas apes and children show continuity in the types of consolation behavior, bonobos are also known to use an array of socio-sexual contacts (e.g. mounting, genital touches, copulation), which are quite unlike what is typical of human infants [14]. As a result, similarity of the underlying mechanisms is not guaranteed.

To date, our understanding of the determinants of non-human consolation comes mostly from studies of chimpanzees [2], [8]–[9], [11]–[13], [22], [34], whereas our other closest living relative, the bonobo, has received little attention [14]. Nevertheless, bonobos are a particularly relevant model species for investigating consolation. Bonobos outperform chimpanzees in experiments related to theory of mind and an understanding of social causality [35]. They are also more tolerant and less aggressive than chimpanzees [36]–[37], and have been called the most empathic ape [38]. Neuroanatomical evidence further suggests that bonobos have more pronounced neural structures for social cognition and empathic sensitivity than chimpanzees [39]. In the current study, we address the scope to which bonobos show consolation and the underlying factors. One aim was to test the familiarity hypothesis, which predicts that third-party affiliation with victims following a conflict is predicted by social affiliation and kinship. Another aim was to address the age trajectory of responsiveness to distressed parties. In contrast to most previous studies, which either excluded immature individuals or did not explore age as a factor [2], [7]–[8], [11]–[13], [34], [40], we included data from individuals across a broad age range, which allowed us to examine the development of consolation in bonobos and test whether consolation requires sophisticated perspective taking skills. If this were the case, we would expect consolation to increase with age along with the increase of such skills.

In addition to examining consolation, we explored conflict resolution between former opponents. Evidence from a broad range of primate species and other social animals has shown affiliative behavior between former opponents following conflict, known as *reconciliation* [1], [9]. Reconciling after conflict is thought to repair bonds with valuable social partners, which provide agonistic support, stress-relief, resource defense, and resource sharing. As described earlier, some studies have suggested an intricate relationship between reconciliation and consolation, with the latter functioning as a form of bystander-mediated reconciliation [4]–[5]. Following the “valuable relationship hypothesis” [41] we predicted that reconciliation would be more likely between kin or closely-bonded opponents.

We explored these patterns in a population of bonobos at the Lola ya Bonobo sanctuary, near Kinshasa. To date, the only data on post-conflict reconciliation and consolation in bonobos comes from studies on small captive groups [14], [42]. Our study site is the largest bonobo facility in the world and thus provides a unique opportunity to collect data from bonobos of mixed age, sex, and rearing history, roaming a semi-free naturalistic environment.

## Results

**Figure 1:**
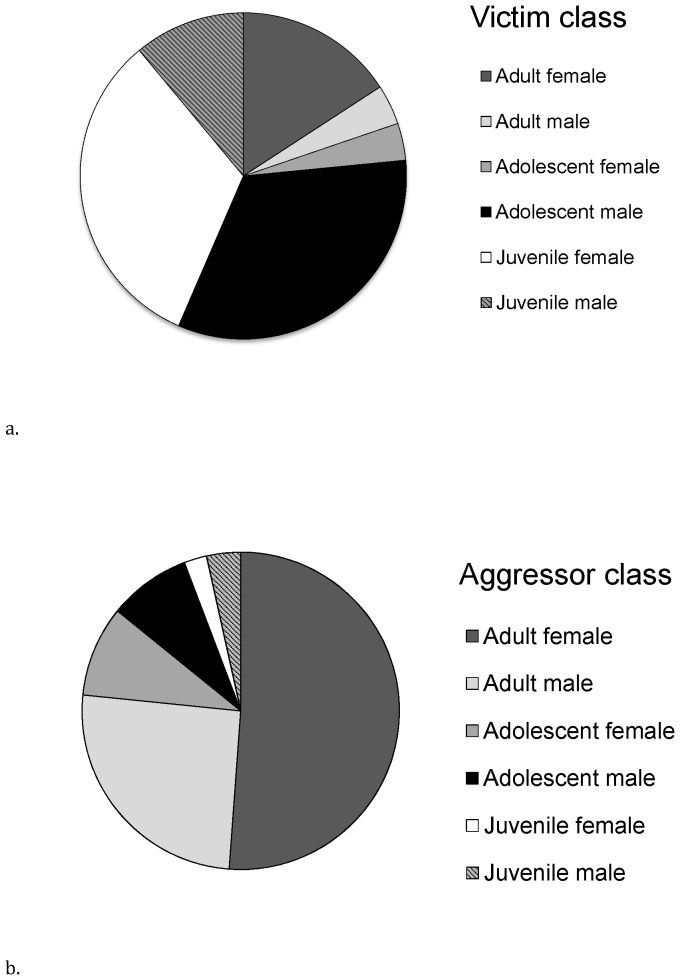
Percentage of agonistic conflicts encountered by different victim and aggressor classes. Pie charts show the percentage of total agonistic conflicts (N = 356) encountered by different victim (a) and aggressor classes (b) in the bonobo population at the Lola ya Bonobo Sanctuary.

A total of N = 356 conflicts were recorded. The distribution of conflict frequency across victim and aggressor classes is shown in Figure 1. Overall, the majority of victims were adolescent males (33.1% of agonistic interactions) or juvenile females (32.5%) whereas adults, particularly females, were the most frequent aggressors (adult females: 51.2%; adult males: 25.4%). The majority of aggressions were medium intensity (chase, shove; 34% of conflicts) to medium high contact intensity (grab, hit, slap; 32% of conflicts) although lower and higher levels were also observed (threats: 14.2%; directed charge display without contact: 2.5%; multiple hit, grab, bite: 12.5%; injurious physical attack/bite: 4.1%)

### Occurrence of consolation and reconciliation

#### Consolation

After excluding cases lacking matched controls, we were able to include 346 PC/MC pairs for analysis. The proportion of attracted pairs was significantly greater than dispersed pairs, indicating that bystanders were providing consolation to victims following conflicts (mean ± SD of the % of attracted pairs = 53.5%±28.2%; dispersed pairs = 20.2%±22.0%, Wilcoxon signed ranks test per focal individual: Z = −3.53, N = 32, P<0.001, two-tailed). As we found very similar patterns across both groups, we were able to combine the data (Table S1 provides separate analyses), without significant differences in the proportions of attracted or dispersed pairs between groups (tested with a Mann-Whitney Test per individual focal, table S1).

As a measure of consolation tendency, we calculated the mean Triadic Contact Tendency (TCT) per victim [6]. The mean TCT levels+SD were 34.74%±35.59 without a significant difference between both groups).

#### Reconciliation

The proportion of attracted and dispersed pairs was compared for affiliative contact between former opponents. We found significant evidence for reconciliation, with the proportion of attracted pairs significantly greater than dispersed pairs (mean ± SD of proportion of attracted pairs = 27.1±22.6%; dispersed pairs = 4.8%±8.9%; Z = −4.29, N = 32, P<0.001, two-tailed). As with consolation, we combined data across groups since we found no significant difference between them (see Table S1). The mean+SD Conciliatory Contact Tendency (CCT) was 22.31%±23.57.

### Latency of post-conflict affiliation

**Figure 2:**
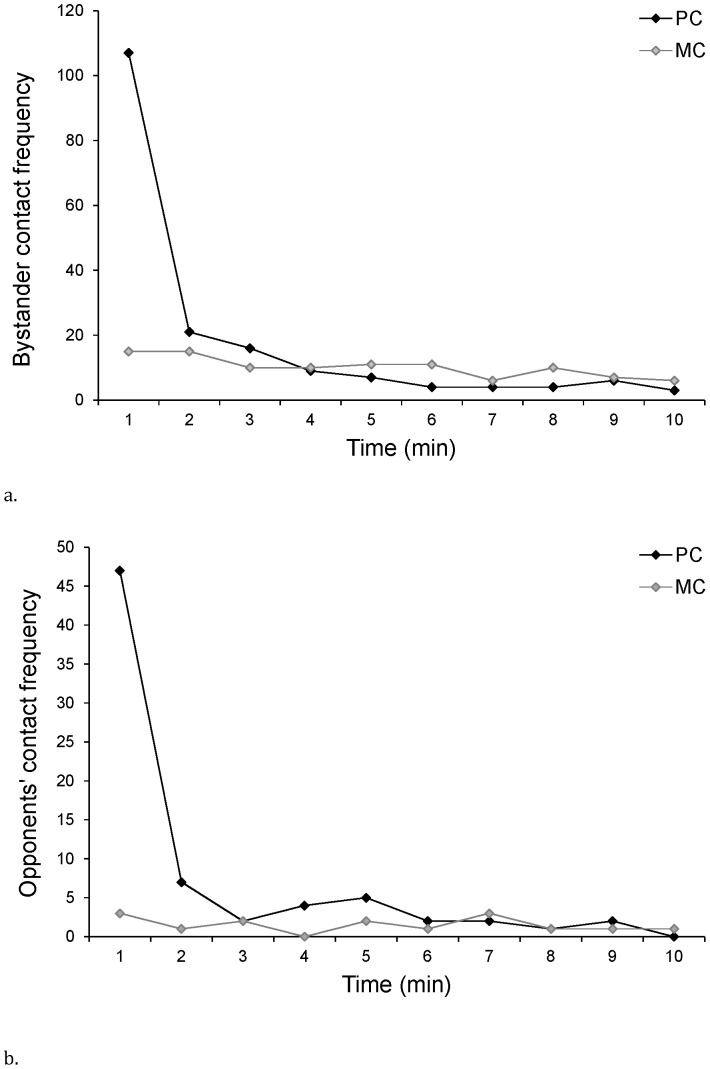
Frequency of first affiliative contacts in conflicts compared to Matched Controls. Frequency over all observations combined of the first affiliative contact offered by (a) bystander to victims of aggression and (b) between opponents in the first ten minutes immediately following conflicts compared to Matched Controls.

We compared the latencies to first affiliative contact in the PC and MC periods. Figure 2 demonstrates a striking peak in both affiliation offered to victims by third-parties (consolation) and between opponents (reconciliation) in the first minute following the conflict as compared to baseline periods. Congruent with Figure 2, a Survival analysis revealed a significant tendency for both bystander-initiated affiliation and between-opponent affiliation to occur earlier in the PC compared to the MC (Kaplan-Meier Survival Analysis: Mantel Cox test for consolation: N = 346 PC/MC pairs, χ^2^ = 50.8, P<0.001; for reconciliation, χ^2^ = 14.3, P<0.001).

### When does consolation occur and who provides it?

A GLMM analysed the factors determining consolation. When all possible models were compared using the AIC, the best fitting model included a combination of non-correlated variables relating to both the conflict itself as well as social variables regarding the bystander and the opponents (AIC = 1894.3, χ^2^ = 4.46, df = 1, P = 0.034; Table 1). The best model fitted significantly better to the data than the null model, which only included random factors (P<0.001).

**Table 1:** The best fitting GLMM model for the occurrence of consolation in bonobos housed at Lola ya Bonobo Sanctuary.

		*AIC*	*X2*	*df*	*P*
		**1894**	**4.458**	**1**	**0.034**
**Fixed Effects**					

Conflict variables	*Levels of factor*	*β*	*S.E*	*Z*	*P*
***Bystander proximity		−0.691	0.101	−6.840	<0 .001
***Redirection		1.134	0.324	3.506	<0 .001
**Context	Non-feed vs feed	0.395	0.135	2.934	0.003
* Reconciliation		0.313	0.145	2.158	0.031
Social variables					
*** Bystander rearing	Orphan vs mother-reared	−1.397	0.344	−4.065	<0.001
*** Victim-bystander affiliation		0.441	0.111	3.968	<0.001
** Bystander-victim kinship	Kin vs non-kin	1.111	0.365	3.044	0.002
* Bystander-aggressor kinship	Kin vs non-kin	0.538	0.255	2.116	0.034
* Bystander age	Juvenile vs adult	0.660	0.297	2.220	0.026
	Adolescent vs adult	0.452	0.269	1.679	0.093
Victim sex	Male vs female	−0.502	0.281	−1.785	0.074
Bystander sex	NS				>0.05
Victim age	NS				>0 .05
Interactions					
***Bystander rearing×victim sex	Mother-reared byst'/F victim	1.060	0.277	3.820	<0 .001
** Victim age×Bystander sex	Juvenile victim/M bystander	1.036	0.374	2.772	0.005
	Adol' victim/M bystander	1.252	0.409	3.061	0.002
* Redirection×Victim age	Adolescent vs adult	−1.054	0.441	−2.386	0.017
	Juvenile vs adult	−0.813	0.393	−2.068	0.039

Random factors	Variance	SD
Post-conflict Interaction number	1.494 e-11	3.866 e-06
Victim identity	0.101	0.319
Bystander identity	0.107	0.327
Aggressor identity+Group	0.000	0.000

Asterisks represent significance values.

*** = P<0.001;

** = P<0.01,

* = P<0.05.

**Figure 3:**
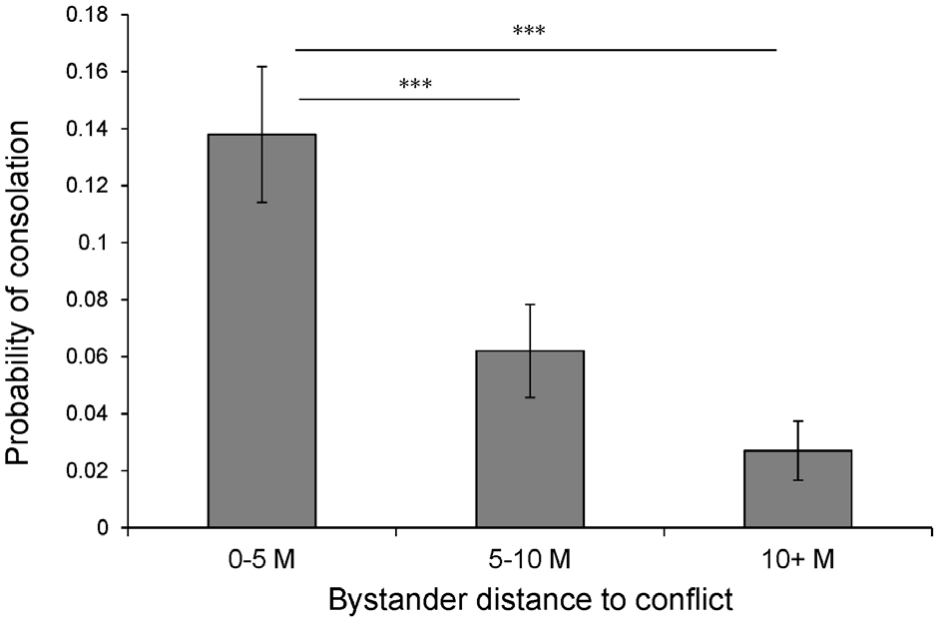
The probability of providing consolation as a function of the bystander proximity to the conflict. Bar chart indicated means+SD. Asterisk indicates P<0.001.

**Figure 4:**
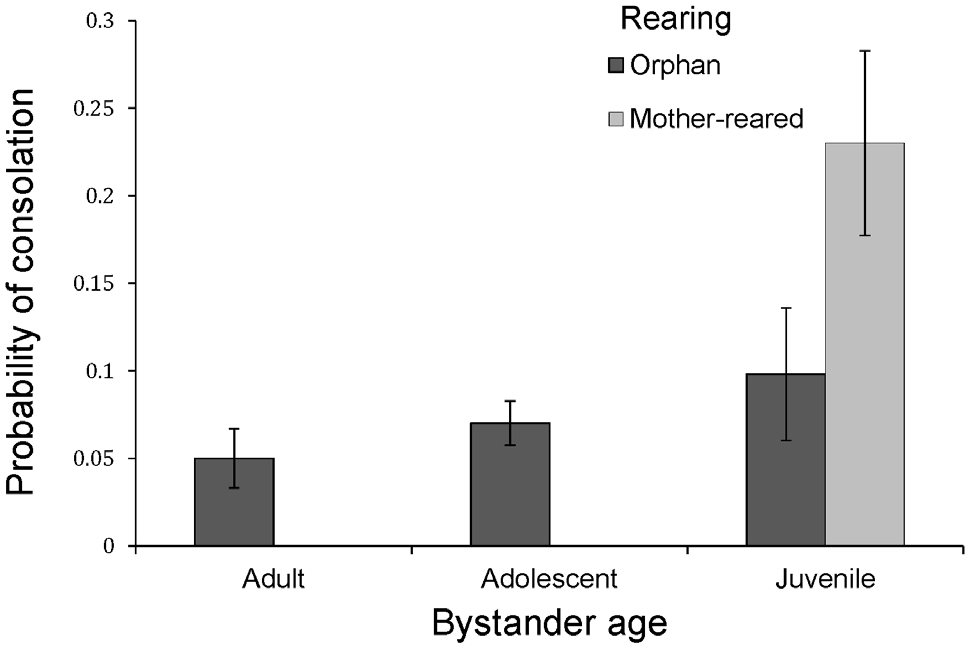
The effect of bystander age and rearing on providing consolation to victims of aggression. The graph provides the mean+SEM proportion of conflicts per individual to which they were bystander. Corresponding GLMM models revealed significant effects of both bystander age and rearing (significant differences between juveniles vs adults, adolescents vs adults, mother-reared vs orphans, see Table 2). The asterisk indicates P<0.05.

Among the variables in the model, there were four that most strongly predicted the occurrence of consolation (Table 1, all P<0.001). The strongest predictor was the distance of the bystander to the conflict, with bystanders in close proximity (<5 m) significantly more likely to console victims than more distal ones (bystander proximity: P<0.001, see Fig. 3). Consolation was also more likely following redirected aggression by the victim towards another bystander other than the consoler (P<0.001). However, there was a significant positive interaction between redirection and victim age that revealed that consolation was only more likely to occur when adult bystanders redirected their aggression (Fig. S1), as compared to adolescent or juvenile victims. We found a strong positive effect of victim-bystander affiliation, showing that bystanders were more likely to console victims with whom they had a close affiliative relationship compared to those with whom they had a weak bond (P<0.001). There was no correlation or interaction between bystander proximity and bystander-victim affiliation. There was a significant effect of bystander age, with juvenile bystanders significantly more likely to console than adults, which did not interact with victim age. There was also a strong effect of bystander rearing, with orphans less likely to provide consolation compared to mother-reared bystanders (P<0.001, Fig. 4). Rather than mother-reared juveniles simply contacting their mothers, perhaps as a form of self-protection, analysis of the types of victims consoled by mother-reared juveniles revealed wide distribution, with mothers receiving only an average of 12.5% of their consolatory contacts (Table S2).

Other significant variables included in the model were the context, the occurrence of reconciliation and bystander kinship to both the victim and the aggressor. Consolation was more likely in non-feeding compared to feeding contexts (P = 0.003) and when opponents reconciled than when they did not (P = 0.031). We found a strong positive effect of kinship between the bystander and the victim (P = 0.002) and to a lesser extent, between the bystander and the aggressor (P = 0.03). While bystander sex and victim age did not significantly contribute to the model alone, there was a significant interaction between them, with male bystanders most likely to console juvenile victims than female bystanders (P = 0.005). There was also a significant interaction between bystander rearing and victim sex, with mother-reared bystanders more likely to console females compared to males (P<0.001).

Despite a clear interaction between bystander age and rearing (Fig. 4), we were unable to directly analyse this interaction in this model because all mother-reared bystanders were also juveniles. Therefore, to examine the effect of the other variables without the influence of bystander rearing, we ran a second, reduced GLMM that excluded mother-reared bystanders (N = 30 bystanders, after removing N = 6). All other features of the model creation and selection remained the same. In this case, the best fitting model looked strikingly similar to the original with strong effects of bystander proximity, context, victim-bystander affiliation levels, victim-bystander kinship, and bystander age (all P<0.001, see Table S3). While both adolescents and juveniles were still more likely to console than adults, the effect of juveniles was less strong (β = 0.53, SE = 0.28, Z = 2.22 P = 0.027) reflecting the influence of mother-reared juvenile bystanders in the original complete model.

#### Reduced Model on Mature Individuals

To compare with previous studies [2], [12], we conducted a reduced GLMM analysis that excluded data from juveniles (N = 190 interactions). Following the model selection procedure, comparison using log-likelihood ratios showed the best fitting model (AIC = 398.5, χ^2^ = 8.50, df = 1, P = 0.0035) still fitted the data significantly better than the null model (P<0.001). The best fitting model was simpler (3 factors, no interactions) but consistent with the main model. We found a strong positive effect of bystander-victim affiliation (β = 0.45, S.E. = 0.220, Z = 2.06, P = 0.039), bystander proximity (β = −0.70, S.E. = 0.28, Z = −2.46, P = 0.014) and to a lesser extent, bystander age, with adolescents more likely to console than adults (β = 0.76, S.E. = 0.393, Z = 1.93, P = 0.053).

### Relationship dynamics and consolation

Linear Mixed Models (LMMs) were used to further investigate the influence of social variables on consolation, using the continuous dependent variables Triadic Contact Tendency (TCT) and the Consolation Index. This analysis was based on dyadic data, that were calculated across all events (i.e. a TCT and Consolation Index score per dyad), which differs from the GLMM analyses that take each individual conflict case, controlling for repeated entries per individual/conflict.

The best fitting model (AIC = 4788.1, χ^2^ = 10.58, df = 0, P<.001), which included two variables, bystander age and bystander kinship, was significantly better at predicting dyadic TCT's when compared to the null model, which included only random effects (P<0.01). Juvenile bystanders consoled significantly more often than adults or adolescents (β = 8.59, SE = 2.76, T = 3.11, P = 0.002), as did bystanders related to the victim compared to non-kin (β = 24.31, SE = 6.45, T = 3.77, P<0.001). Bystander affiliation and bystander rearing also had significant predictive effects but were removed from the best fitting final model owing to a significant correlation with kinship.

Using the Consolation Index as a measure of consolatory tendency, we found that the best fitting model (AIC = 4295, χ^2^ = 3.92, df = 1, P = 0.047) included thee fixed effects: bystander-victim kinship, bystander's rearing and bystander sex. Victims were more likely to be consoled by bystanders that were kin (β = 35.270, SE. = 4.067, T = 8.673, P<.001); mother-reared (β = 11.196, SE = 2.355, T = 4.754, P<.001) and male, although sex just failed to reach significance (β = 3.441, SE = 1.805, T = 1.906, P = .057). As with TCT, we found significant correlations between factors, which forced us to exclude bystander age (as it was correlated with bystander rearing), although it was a significant factor in competing models.

In sum, our combined results from the LMM analyses indicate that consolation was most likely to be provided by bystanders that were juvenile; that share either a close affiliative or kin bond with the victim and that have been mother-reared.

### Effect on victim stress

**Figure 5:**
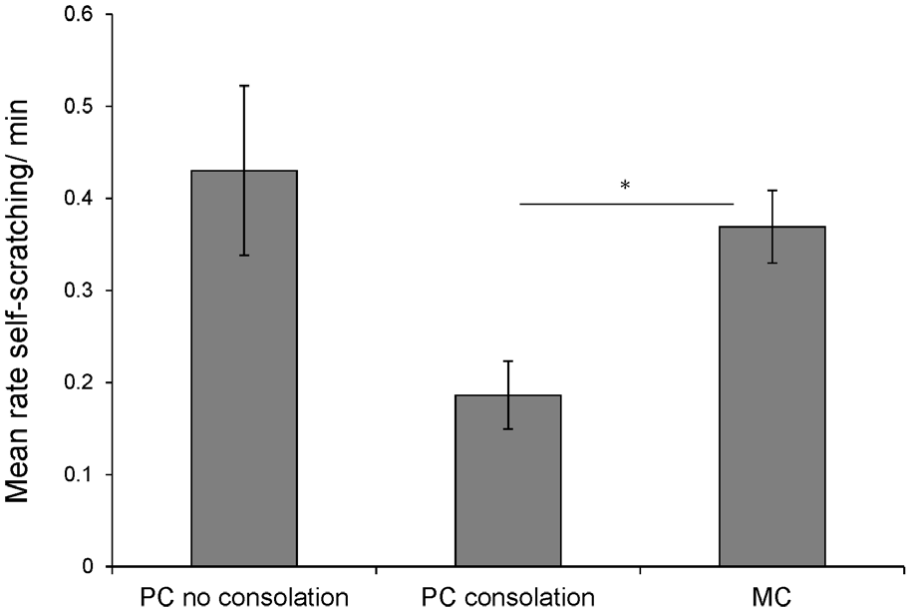
Rates of victim self-scratching during Post-Conflict periods with or without consolation compared to Matched Controls. The bar chart indicates mean ± SEM rates of self-scratching by victims during Post-Conflict and (PC) and Matched Control periods (MC). To remove the confounding influence of reconciliation, PCs with reconciliation were removed from analysis. The asterisk indicates P<0.05.

Mean rates of self-scratching and mean durations of self-grooming during PC/MC periods were compared to examine whether consolation had a stress-alleviating effect, see Fig. 5 [12]. The distribution of PCs types were: Consolation alone: N = 146; Reconciliation alone: N = 34; Consolation+Reconciliation: N = 56; No affiliation: N = 110. Baseline (MC) levels of self-scratching found were higher in this population of bonobos compared to previous studies of chimpanzees using similar methodologies [2], and we found that rates of self-scratching during post-conflict periods without affiliation (PC) were not significantly higher compared to baseline (MC) (Mean ± SD rate in MC = 0.37±0.22; PC = 0.43±0.45; Wilcoxon signed-ranks test Z = −0.26, N_1_ = 32, N_2_ = 24, NS; Fig. 5, Table S4). However, self-scratching significantly decreased in PCs in which consolation occurred (Mean ± SD in PCs with consolation = 0.19±0.19; Wilcoxon signed-ranks test comparing with the MC rate: Z = −3.99, N_1_ = 32, N_2_ = 29, P<0.001). Rates of self-scratching decreased in PCs with consolation compared to PCs without, but the result just failed to reach significance (Z = −1.981, P = .048; Fig. 5), after implementing the Bonferroni correction (α = 0.016). To examine whether contact generally has a stress-reducing effect that is not specific to conflicts, we examined rates of self-scratching in MCs in which the focal individual did or did not receive contact affiliation. Unlike the PCs, we found no significant drop in scratching rate for contact vs. no contact MCs (Wilcoxon signed ranks: Z = −0.037, N = 20, P>0.05).

Baseline levels of self-grooming (mean duration per min) did not differ significantly from PCs without affiliation (Mean ± SD sec per min in MC = 3.88±6.12; PC without affiliation = 2.90±3.71; Wilcoxon signed-ranks test Z = −0.40, N_1_ = 32, N_2_ = 25, NS; Table S4). However, as with self-scratching, we found a reduction in self-grooming in PCs with consolation as compared to MCs, which just failed to reach significance (Mean+SD duration per minute in PC with consolation = 1.98s±2.66; Z = −1.78, N_1_ = 32, N_2_ = 28, P = .075). There was no significant decrease in self-grooming in PCs with consolation compared to those without.

### Reconciliation

GLMM analyses examined the factors predicting reconciliation. When all possible models were compared using the AIC, the best fitting model included four fixed effects and no interactions (AIC = 354.83, χ^2^ = 9.87, df = 2, P = 0.007). Only variables relating to opponent type and affiliation were retained. Reconciliation was positively predicted by the degree of affiliation between opponents (β = 0.505, P = 0.04) as well as the ages of both opponents, with juvenile aggressors more likely to reconcile than adolescents or adults and adolescent victims more likely to reconcile than adults or juveniles, see Table 2). Similar to consolation, there was a significant effect of rearing, with mother-reared victims more likely to reconcile than orphaned ones (β = −1.25, P = 0.013).

**Table 2:** Best fitting GLMM model for the occurrence of reconciliation.

		*AIC*	*X2*	*df*	*P*
		**356.5**	**10.481**	**0**	**<0 .001**

Fixed Effects	*Levels of factor*	*β*	*S.E*	*Z*	*P*
**Victim-bystander affiliation		0.505	0.175	2.893	0.004
* Victim age	Juvenile vs adult	−0.871	0.542	−1.609	>0.05
	Adoles vs adult	0.848	0.441	1.924	0.054
*Victim rearing	Orphan vs mothered	−1.249	0.505	−2.475	0.013
**Aggressor age	Juvenile vs Adult	1.764	0.581	3.036	0.002
	Adolescent vs adult	0.345	0.355	0.971	>0.05

Random Factors	Variance	SD
Post-conflict interaction	0.000	0.000
Victim identity	0.071	0.266
Aggressor identity	6.11 e-10	2.47 e -05

Asterisks represent significance values:

*** = P<0.001;

** = P<0.01,

* = P<0.05.

## Discussion

Bonobos across age and sex classes spontaneously offered consolation to distressed parties. Their behavior appeared to alleviate the victim's stress as indicated by a drop in self-scratching following consolation compared to both baseline and post-conflict periods without consolation. No such drop occurred after affiliative contacts during matched controls. A global analysis of the determinants of consolation revealed physical proximity of the bystander as the strongest predictor, with nearby bystanders significantly more likely to contact victims than more distal ones. Proximate mechanisms of reducing stress in others therefore reflect simple pragmatics of being physically close. Nevertheless, this obviously cannot be the full explanation, since most monkey species lack active consolation despite often being close to conflict.

Not all nearby bystanders consoled victims and our model revealed a number of additional determining factors. Bystanders were significantly more likely to console relatives or closely bonded partners, a result that carried across subsequent analyses and was independent of the physical proximity implied in close relationships. The effects of social closeness are consistent with studies on chimpanzees [12]–[13] and are congruent with an empathy-based explanation, with similarity, familiarity and social closeness considered to facilitate empathy in both humans and other animals [24], [43]–[44]. Partners sharing stronger affiliative bonds are more likely to be sensitive to each other's distress. Therefore, consolation may serve to reduce the distress not only of the victim, but also of bystanders tuned into the victim's emotional state. However, a number of alternative mechanisms may go some way to explain this effect (such as responses to aversive stimuli, association learning, fear of retaliation, and gaining reciprocal support) and should be addressed in future work. Observational studies, such as ours, cannot easily differentiate between underlying mechanisms, but they nevertheless provide crucial information for the development of experimentally testable predictions.

Affiliation levels also positively predicted the occurrence of reconciliation, with closely bonded former opponents more likely to reconcile than weakly bonded ones. Associations with closely bonded partners may confer considerable fitness benefits to both parties (i.e. agonistic support/food sharing/resource defense), hence repairing relationships with these individuals – known as the “Valuable Relationships Hypothesis” [41] – is considered particularly important [1].

That consolation was predicted by affiliation between bystander and victim, and *not* bystander and aggressor, contradicts the hypothesis that consolation acts as a substitute for reconciliation on behalf of the aggressor [4]–[5]. Furthermore, we found a significant predictive effect of reconciliation on subsequent consolation, which is the opposite of what one would expect under the Substitution hypothesis (i.e. if consolation replaces reconciliation, one would expect to see it more if reconciliation has not occurred). While the strongest kinship effect occurred between bystanders and victims, the possibility that bystanders console on behalf of aggressors cannot entirely be ruled out, as we still found a weak but significant effect of bystander-aggressor kinship on the occurrence of consolation

Neither did we find strong evidence for the Protection Hypothesis. While the likelihood of consolation went up in association with redirected aggression, the fact that consolation was mostly provided by individuals socially close to the victims contradicts the hypothesis that they were trying to protect themselves. The typical victims of redirected aggression were not the most frequent consolers and did not console following redirected aggression. Adult female victims were most likely to redirect aggression, and their targets were typically adolescent males and juvenile females (Fig. S1). The tendency to re-direct onto (mostly orphaned) adolescent males and juvenile females may reflect the low social status of immature individuals lacking maternal support in the sanctuary setting. Maternal support is a particularly relevant issue in bonobo society, where mothers maintain high-status positions and support their offspring in fights [37]–[38]. The possibility that self-protection motivated the strong consolatory tendencies of mother-reared juveniles was also unlikely, because mother-reared juveniles offered consolation to a wide range of victims, with their mothers only representing a very small minority of their consolation targets (Table S2).

While our results are most consistent with the consolation hypothesis, other explanations may also help to explain why bystanders spontaneously contact victims in certain cases, as different functions and mechanisms need not be mutually exclusive (for instance, associative learning, responses to aversive stimuli and to a lesser extent substitution for reconciliation). Even within the same species, such as the chimpanzee [7], [12]–[13], [34], the literature indicates considerable flexibility in the functions of bystander affiliation towards victims, which can vary depending both on the context and the social conditions.

A key finding in our study was the effect of bystander age, revealing that consolation was more likely to be offered by younger bystanders, especially juveniles compared to adults. This was maintained in a reduced model where an interacting factor, bystander rearing, was removed. Most previous post-conflict studies have either excluded immature subjects or not explicitly investigated age effects [6]–[8], [12]–[14], [45], possibly because juveniles were either assumed not capable of effectively consoling others or were deemed irrelevant in relation to the relationship network. Our finding that juveniles consoled more than adults challenges the assumption that consolation necessarily requires an advanced cognitive overlay that emerges only with age. Rather, consolation seems to emerge early on via mechanisms that may be simpler than sometimes assumed.

In human infants, sympathetic concern for others increases across the second year, concurrent with the onset of a suite of related skills in the domains of inhibition, emotion processing, emotion regulation and appraisal of others' emotional and mental states [25]–[27], [30]–[31], [46]–[48]. However, the first signs of sensitivity to the emotional states of others and expressions of cognitive empathy appear at an earlier age [25], [27]–[29]. While this sensitivity has traditionally been considered to be more self-orientated, more recent evidence is challenging this assumption and indicates that empathic concern may reflect fairly simple forms of self-other differentiation [27], [48]. In a developmental study, personal distress reactions by 8–16 month old infants to others' distress were rare but modest forms of cognitive empathy were already present [27]. Although we currently have little information about how these processes develop in other animals, our study suggests that juvenile bonobos are already able to reduce victim distress and respond to their emotional states. Whether the nature of bonobo consolation behaviors also changes with age (i.e. whether the nature of juvenile consolation behaviours differs to that offered by adults) and whether or not bonobos display the propensity to console prior to the age range of our juveniles (from 3 to 7 years) will need to be addressed by future work that also includes infants.

The finding that juvenile bonobos console victims also fits the notion of ‘pre-concern’, a hard-wired building block that is thought to emerge before the onset of more advanced forms of sympathetic concern [47]. Pre-concern goes beyond personal distress and the alleviation of self-distress in that it is other-oriented and reduces someone else's distress, but without necessarily comprehending their specific situation. This may mirror some of the forms of empathic responding seen in young infants below the age of two years, who appear to possess modest levels of affective and cognitive empathy [27]. Nevertheless, cross-species generalizations must be treated with care, and high levels of socio-sexual forms of consolation behavior by bonobos suggest a key difference with humans [14]. It will also be important to identify whether more sophisticated forms of concern in non-human primates develop over time, and if so, whether they mirror changes observed in human infants.

Mother-reared individuals were significantly more involved in post-conflict interactions than orphans. First, when themselves in the victim role, mother-reared individuals more often reconciled with aggressors. Second, as bystanders to conflict, they were more active consolers of victims. Both findings highlight the role of rearing and early attachment in emotional development, and suggest that individuals who have been reared in a species-typical way by their own species are better equipped to both comfort others and to reconcile conflicts when these arise. Since Harlow and colleagues [49]–[51], it has been acknowledged and re-demonstrated [52] that maternal care in infancy is critical for the development of secure and organized attachment styles as well as for cognitive and socio-emotional development. Our study is consistent with this framework, and is congruent with studies of human infants, which indicate that empathy and emotion regulation are negatively impacted by early trauma, deprivation and disruptions in development [32], [53]–[55]. Alternatively, this rearing effect could suggest that, compared to orphans, mother-reared individuals have had more opportunities to socially learn and associate their actions with situational outcomes. Mother-reared individuals also benefit from the support of their mothers, which may consequently influence their temperament or willingness to approach others in distress. The presence of the mother is particularly important in bonobo societies, where females have dominant social positions, often dominate males and maintain long-term relationships with their sons throughout adulthood [37]–[38], [56]. Overall, our study has highlighted the relevance of rearing experiences for interpreting social behaviour and has laid out some novel developmental approaches to the study of post-conflict interactions in animals. Future work will need to further address the role that rearing plays in responsiveness to distress and the developmental trajectory of consolation behaviours.

## Methods

### Ethical statement

Permission for this observational study came from ‘Les Amis des Bonobos du Congo’ (ABC) following full ethical approval from Les Amis de Bonobos du Congo (ABC) Scientific Committee and its Scientific Coordinator. It complied with all legal requirements required for conducting research in DR Congo. This study fully complied with Emory's IACUC guidelines for conducting observational studies.

### Study Site and Subjects

Observations of bonobos were conducted at the Lola ya Bonobo Sanctuary, Kinshasa, DR Congo. Most individuals arrive at the sanctuary as wild-caught infant or juvenile orphans as a result of the bush-meat and pet trades. Following several years of rehabilitation within a nursery “cohort group,” where each individual is assigned a substitute human mother, individuals are integrated into large, mixed-age social groups. A number of offspring have also now been born at the Sanctuary, which are also included in the data set (see Table 3). Individuals spent their days ranging outdoors in one of three naturalistic forest enclosures (15–20 ha), which were comprised of rainforest, lake, swamp, streams and open grass areas. At night, individuals slept together inside dormitories (approx. 75 m2, divided into open sub-rooms). We collected data when the bonobos were in the visible areas of the enclosure. The bonobos were provisioned 3–4 times per day by caregivers with a variety of fruits and vegetables. Their daily routines remained the same throughout the observation period.

**Table 3:** Composition of study groups, housed at Lola ya Bonobo Sanctuary, DRC.

Group 1				Group 2				
Name	Code	Age	Age class	Name	Code	Sex	Age	Age class
**Females**				**Females**				
Opala	OP	16	A	Maya^+Mayele(m)^	MY	F	18	A
Semendwa^+Makasi(m)^	SW	14	A	Tshilomba^+Sanza (m)^	TL	F	20+	A
Bandundu	BD	14	A	Isiro	IS	F	13	A
Kalina^+Bolingo (m)^	KL	13	A	Likasi	LI	F	10	AD
Salonga^+Kimia (f)^	SL	13	A	Sake	SK	F	6	J
Kisantu^+Liyaka(f)^	KS	12	A					
Lisala	LS	10	AD					
Katako	KT	7	J					
Elikia*^(SW)^	EK	6	J					
Masisi	MS	5	J					
Waka	WK	5	J					
Malaika*^(KL)^	ML	4	J					
**Males**				**Males**				
Manono	MN	17	A	Keza	KZ	M	20+	A
Kikwit	KW	13	A	Makali	MK	M	20+	A
Fizi	FZ	12	A	Max	MX	M	25	A
Matadi	MA	11	AD	Lomami	LM	M	12	A
Dilolo	DL	10	AD	Mbandaka	MB	M	9	AD
Kasongo	KG	9	AD	Bili	BL	M	10	AD
Mabali	MB	8	AD	Ilebo	IB	M	9	AD
Pole*^(OP)^	PO	6	J	Yolo	YL	M	7	AD
Wongolo*^(BD)^	WO	3	J	Bisengo*^(MY)^	BS	M	6	J
				Moyi*^(TL)^	MO	M	4	J

Age classes are indicated by A = Adult, AD = Adolescent, J = Juvenile. Asterisks indicate mother-reared individuals born at the sanctuary with the identity of their mother in superscript. The parentheses indicate the sex of the offspring (m = male; f = female).

We conducted observations at enclosure 1 (Group 1) and enclosure 2 (Group 2). Group 1 comprised of 25 individuals (6 adult females, 3 adult males and 16 immatures) and Group 2 comprised of 17 individuals (3 adult females, 4 adult males and 10 immatures). For more details see Table 3. As exact birth dates for orphaned sanctuary apes are generally unknown, we used age estimates made by sanctuary veterinarians upon arrival (typically, between 1–3 years old), which were adjusted based on measurements of weight and patterns of dental emergence according to known patterns of ape development [57]–[58]; Wobber & Rosati, Pers. Comm. This technique was validated by the known exact ages of individuals born at the sanctuaries, which we also used.

### Data Collection

From May–August 2011, observations of Groups 1 and 2 were conducted by Z.C and an assistant throughout the day, with a total of 301 and 152 observation hours recorded at Group 1 and Group 2, respectively. We conducted all-occurrence observations of agonistic interactions that included at least one of the following behavioral elements: recipient fleeing and/or screaming in reaction to aggression, and aggressor threat barks/grunts, directed display charge, threat arm wave, chase, hit, trample, slap, shove, poke, or bite. For each agonistic interaction, we recorded the identities of the initial recipient of the aggression, which we will call the “victim,” and the aggressor, as well as the identities of all visible bystanders. We recorded bystander proximity at three levels: bystanders within 5 m of the conflict, those between 5–10 m and those beyond 10 m. For each interaction, we also recorded the conflict context (i.e. feed, rest, play, object/food competition (physically disputing a specific food item or non-food object i.e. branch), arrival (an individual or group of individuals join the group, i.e. after a later release from the dormitories); anticipating feed (<15 min prior to feed arrival), social tension/display) and the conflict intensity, which ranged from (1) threat (hand shake, bipedal swagger threat/whistle bark, lunge); (2) directed display/charge without physical contact; (3) chase pursuits or quick poke/shove; (4) single grab/hit/slap without biting; (5) severe/multiple grab/hit or biting; and (6) injurious physical attack or biting [59].

For each interaction, we conducted focal sampling of the victim using the standardized Post Conflict (PC)-Matched Control (MC) method [47]. For post-conflict focals (PC), this consisted of a 10-minute focal sample of the victim immediately following the conflict interaction. Each PC was matched with a 10-minute Matched Control (MC) focal, which was conducted on the same victim, the following day (±2 days) at the same or closest possible time (±1 hr). MC s were only conducted if both the victim and the aggressor were present within 10 m of one another and if the focal individual had not been involved in a conflict interaction for at least 10 min prior to the MC. If the focal had been involved in a conflict interaction within 10 min, the MC was postponed for at least 10 minutes after the end of the conflict, for up to one hour after the scheduled MC time. As with PCs, we recorded the presence of all visible bystanders and their physical proximity to the focal individual (<5 m, 5–10 m, >10 m). During PC and MCs, we recorded all instances of affiliative contacts between the focal individual and the original opponent or with any other bystander. Affiliative contact behaviors included embrace, socio-sexual contact (i.e. genito-genital contact, mount, copulation, genital touch), touching, grooming, contact sitting, play, hold, pat and inspect (see List S1 for more detail). We also recorded the initiator of each interaction, which was the individual starting the interaction.

In addition to affiliative contacts, we also collected data on levels of self-directed behaviors in PC and MC periods by recording rates of self-scratching per minute and durations of self-grooming across the focal period [2]. All focals were filmed using a Canon Vixia HF200 HD Camcorder. Aside from interactions involving dependent infants, interactions involving all individuals in the both groups were included in the analyses.

In order to construct affinity matrices, instantaneous scan samples of all visible individuals in the group were carried out throughout the day, with a minimum of 10 minutes between scans. At each scan, the identities of all visible party members were recorded followed by the identities of all individuals engaging in one of the following activities: grooming, contact sitting, sitting within arms reach, play or sexual contact (data on these group-level state behaviors are distinct to the focal data collected during the Post-conflict or Matched control focals) Across the study period, we collected a total of 794 and 411 scans at Groups 1 and 2 respectively. Interactions between all individuals, except dependent infants, were recorded.

### Data analysis

#### Occurrence of consolation and reconciliation

We used the PC-MC comparison method in order to detect the occurrence of consolation and reconciliation [60]. For consolation, we considered the first contact affiliations initiated by a bystander towards the victim. For reconciliation, we considered the first contact affiliations between the victim and aggressor. For both reconciliation and consolation, we labeled PC-MC pairs as ‘attracted’ if the affiliation initiated between opponents or by the bystander towards the victim occurred earlier in the PC than the MC, or only in the PC. We labeled PCMC pairs ‘dispersed’ if the affiliation occurred earlier in the MC than the PC, or only in the MC. We labeled PCMC pairs ‘neutral’ if the affiliation occurred at the same time in both the PC and MC or in neither.

To evaluate the occurrence of reconciliation and consolation, we used Wilcoxon signed ranks tests to compare the proportion of attracted and dispersed PC-MC pairs per focal victim. Following Call et al. [6], we also calculated the mean individual Triadic Contact Tendency for conciliatory contacts towards the victim as follows: 100*(Attracted pairs-Dispersed Pairs)/(Total PC-MC pairs); for the first affiliative contact from a bystander to a given victim). For reconciliation, we used the equation above to calculate the Conciliatory Contact Tendency (CCT), but rather used the attracted and dispersed pairs occurring between opponents.

#### Latency of post-conflict affiliation

The latency to provide post-conflict affiliation in PCs was compared to affiliation in MCs using a Kaplan-Meir Survival Analysis with a Mantel-Cox test [18]. The Survival Analysis takes into account ‘censored’ data, which in this case, were any PC or MC focals in which no bystander-initiated affiliation occurred before the end of the observation.

#### What determines when consolation occurs and who provides it?

We used generalized linear mixed models (GLMM) with binomial error structure and a logit link function to find out which partners provided consolation. We explored factors relating both to the conflict (conflict intensity, context, occurrence of reconciliation & redirection) and an array of social variables concerning the partners (victims, aggressors and bystanders). Unlike previous studies, which typically only include mature individuals in the analyses, we included individuals from the point of juvenility, using age as one of the predictor variables. We also looked at the effects of the following variables: sex, kinship (mother-offspring kinship or none), rearing type (orphan versus mother-reared) and degree of affiliation between bystanders, victims and aggressors, using dyadic affiliation scores. We calculated levels of affiliation per dyad using a combined measure of five affiliation behaviors (grooming, contact sitting, sitting within arms reach, play or sexual contact) taken during the scan samples that occurred between a given dyad, divided by the number of scans in which they were both present.

We conducted generalized linear mixed models (GLMM) using the ‘lmer’ function in the R package ‘lme4’. The binomial dependent variable was the occurrence of consolation (yes/no). We operationally defined consolation as being when the first bystander-initiated affiliation towards a victim was ‘attracted’, that is to say it occurred sooner in the PC as compared to the Matched Control (MC). The offering of consolation was entered for each potential bystander for each post-conflict interaction (ie. each post-conflict opportunity of offering consolation was entered as a data point into the model), based on prior comparison per bystander during the matched control (N = 346 interactions). All fixed effects originally entered into the GLMM analyses are shown in Table S5, although we only present here the effects of variables present in the best model (see below for the criterion used).

In order to control for variable bystander presence (i.e. not every bystander was present for every PC and MC), we only included bystanders in the GLMM analysis that were present in both the PC and the MC periods for a given interaction. We log-transformed affiliation measures so that they approximated a normal distribution. We found no strong co-linearity among predictor variables so were able to enter all possible combinations of factors until we found the optimal model to predict consolation. We controlled for repeated sampling and inter-individual/group variation by including five random effects into the model: the identities of Victim, Aggressor, and Bystander; Group; and Post-conflict interaction number. We entered post-conflict interaction number and the identities of victims, aggressors and bystander as random effects in order to control for repeated entries across and within conflict interactions.

We computed all possible models using different combinations of predictor variables and the best model was selected using the Akaike's information criterion (AIC). The AIC compares the adequacy of multiple models and identifies the most parsimonious model that best explains the variance of the dependent variable, while penalizing for the number of variables in the model. The best model, which has the lowest AIC value, is the best model to predict values of the dependent variable in a new data set [61].

#### A Reduced model on Mature Individuals

In addition to the main analysis, we also conducted an analysis on data from mature individuals only (adults/adolescents), which allowed direct comparison with previous studies, which typically exclude juveniles. We conducted the same GLMM analyses as above (N = 190 interactions), and selected the best fitting model using the AIC criterion.

#### The influence of relationship dynamics on consolation

We explored the influence of various social variables on the likelihood of a given bystander to console a given victim. We used the Triadic Contact Tendency (TCT) [6], for each possible victim-bystander dyad, as a measure of post-conflict affiliation. While TCT controls for baseline levels of affiliation, it is based only on the first affiliative contact between that victim and bystander, and so does not account for preceding affiliations that may have occurred with another bystander. To overcome this issue, we also calculated the Consolation Index [12], which is calculated as the frequency each bystander is the f*irst* individual to provide consolatory contact, divided by the number of opportunities that the bystander had to contact that victim. The Consolation Index thus controls for the potential effect of consolation by multiple bystanders although unlike the TCT, does not control for baseline affiliation levels. Therefore, using both complementary measures enables us to account for the effects of multiple consolations as well as for baseline affiliation levels.

We conducted Linear Mixed Models (LMMs) using the ‘lmer’ function in the R package ‘lme4’ to examine the effect of following predictor variables on the TCT and Consolation Index: the sex, age and rearing of the bystander/victim/aggressor, and bystander-victim affiliation (log-transformed owing to data scew). We included the identities of the victim and bystander as random variables as well as the study group. All possible models were compared using the AIC to identify the best model. The significance of each predictor variable in the best model was then calculated using a Markov chain Monte Carlo simulation of 10 000 iterations. To test for overall significance of the fixed effects in the best model, we also conducted likelihood ratio tests comparing the full model (random and fixed effects) with the respective null model (only random effects).

#### Does consolation have a stress-reducing effect?

We examined the occurrence of self-directed behaviors of victims during PC and MC periods in order to examine whether consolation had a stress-alleviating effect [2], [12]. We analysed rates of self-scratching (number of bouts per min) and duration of self-grooming (mean duration per min), measures which have both been used in other post-conflict studies [2], [12], [62]. We used Wilcoxon Signed Ranks tests to compare mean rates of self-scratching and duration of self-grooming in PC's with and without consolation (using the same definitions of consolation as described), as well as comparing both to baseline periods (MCs). For both PCs with and without consolation, we excluded any cases where reconciliation also occurred, in order to control for its potentially confounding effect.

All GLMM and LMM analyses were run using R statistical software (R Core Development Team 2012) and all non-parametric statistical analyses were conducted using SPSS (v19). For non-parametric analyses, we controlled for multiple comparisons using the Bonferroni correction. All analyses were two-tailed and, aside from where the Bonferonni correction was applied, the significance level was set to 0.05.

## Supporting Information

List S1 **List of definitions of affiliation behaviours.**
(DOCX)

Table S1 **Fixed and random factors entered into the GLMM analyses for occurrence of reconciliation.**
(DOCX)

Table S2 **Separate group analyses for Wilcoxon signed-rank tests of consolation and reconciliation.**
(DOCX)

Table S3 **Best fitting GLMM for the occurrence of consolation when mother-reared individuals were excluded.**
(DOCX)

Table S4 **Mean levels of self-directed behaviours in PCs with and without consolation, as compared with MCs.**
(DOCX)

Table S5 **Percentage of consolatory contacts offered by mother-reared juveniles to their mothers.**
(DOCX)

Figure S1 **Pie chart showing the % distribution of victim types that were targets of re-directed aggression during post-conflict interactions.**
(TIF)
