# Supplementary material for: Bonobos Respond to Distress in Others: Consolation across the Age Spectrum
Source: PLoS One. 2013 Jan 30;8(1):e55206. doi: 10.1371/journal.pone.0055206 (PMC3559394; doi:10.1371/journal.pone.0055206)
Supplement: List S1 — List of definitions of affiliation behaviours. (DOCX) [file pone.0055206.s001.docx]

**List S1. List of definitions of affiliation behaviours**

**Embrace:** Individual places one/both arms around the recipient’s body while facing the partner or in lateral position

**Genito-genital contact**: Individuals embrace ventro-ventrally and swing their hips laterally, while keeping their vulvae in contact

**Mount:** Individual makes lateral contact of their genitals behind the recipient and thrusts their pelvis onto the recipients behind/back area.

**Copulation**: Penile intromission and hip thrusting with male or female partner

**Genital touch:** Touching the genitals of the recipient using any body part other than the genitals. I.e. using hand/foot to touch recipient’s penis/genitals

**Touching**: any instantaneous soft touch to the recipients body, other than to their genitals, using any body part other than the actors genitals.

**Grooming**: Directed cleaning/touching/visual inspection of the recipient’s hair or skin

**Contact sitting:** Sitting in physical contact with the recipient, i.e. contact of leg on their back.

**Play:** Individuals wrestle/run/jump/chase/tickle accompanied by play face and/or laughing

**Hold:** grasping/holding onto recipient’s body or hair with one or both hands. Generally when recipient is walking or standing.

**Pat:** Pronounced tapping/patting contact onto recipients body using flat hand

**Inspect**: visual and contact inspection of the recipients wound or injured body part, following a conflict
