# Supplementary material for: Bonobos Respond to Distress in Others: Consolation across the Age Spectrum
Source: PLoS One. 2013 Jan 30;8(1):e55206. doi: 10.1371/journal.pone.0055206 (PMC3559394; doi:10.1371/journal.pone.0055206)
Supplement: Table S1 — Fixed and random factors entered into the GLMM analyses for occurrence of reconciliation. (DOCX) [file pone.0055206.s002.docx]

**Table S1.** Separate group analyses for Wilcoxon signed-rank tests of consolation and reconciliation using mean proportion of attracted and dispersed PCMC pairs

| Group |  | proportion % | SD | Wilcoxon Z | N | P |
| --- | --- | --- | --- | --- | --- | --- |
|  | *Consolation* |  |  |  |  |  |
| 1 | Attracted pairs | 51.30 | 27.36 | -2.797 | 20 | 0.005 |
|  | Dispersed pairs | 19.75 | 20.84 |  |  |  |
| 2 | Attracted pairs | 57.17 | 30.34 | -2.077 | 12 | 0.038 |
|  | Dispersed pairs | 20.83 | 24.74 |  |  |  |
| *Mann Whitney: proportion A pairs Group 1+2* | | | | U | N | P |
|  | | | | 102.5 | 20,12 | > 0.05 |
| *Mann Whitney: proportion A pairs Group 1+2* | | | | U | N | P |
|  | | | | 105 | 20,12 | > 0.05 |
| *Mann Whitney on TCTs for Group 1+2* | | | | U | N | P |
|  | | | | 112 | 20,12 | > 0.05 |
|  | Reconciliation |  |  |  |  |  |
| 1 | Attracted pairs | 24.31 | 17.56 | -3.622 | 20 | < 0.001 |
|  | Dispersed pairs | 2.29 | 4.056 |  |  |  |
| 2 | Attracted pairs | 31.71 | 29.55 | -2.371 | 12 | 0.018 |
|  | Dispersed pairs | 8.91 | 12.89 |  |  |  |
| *Mann Whitney on CCT for Group 1 + 2* | | | | U | N | P |
|  | | | | 98.5 | 20,12 | > 0 .05 |
