# Supplementary material for: Bonobos Respond to Distress in Others: Consolation across the Age Spectrum
Source: PLoS One. 2013 Jan 30;8(1):e55206. doi: 10.1371/journal.pone.0055206 (PMC3559394; doi:10.1371/journal.pone.0055206)
Supplement: Table S2 — Separate group analyses for Wilcoxon signed-rank tests of consolation and reconciliation. (DOCX) [file pone.0055206.s003.docx]

**Table S2.** Percentage of consolatory contacts offered by mother-reared juveniles to their mothers

| Identity | % of contacts offered to mothers | N |
| --- | --- | --- |
| BIS | 0 | 10 |
| EK | 0 | 29 |
| PO | 1.53 | 65 |
| MOY | 12.5 | 8 |
| MLK | 52.94 | 17 |
| WO | 8.10 | 37 |
