# Supplementary material for: Bonobos Respond to Distress in Others: Consolation across the Age Spectrum
Source: PLoS One. 2013 Jan 30;8(1):e55206. doi: 10.1371/journal.pone.0055206 (PMC3559394; doi:10.1371/journal.pone.0055206)
Supplement: Table S3 — Best fitting GLMM for the occurrence of consolation when mother-reared individuals were excluded. (DOCX) [file pone.0055206.s004.docx]

**Table S3**. Best fitting GLMM for the occurrence of consolation when mother-reared individuals were excluded.

|  |  | *AIC* | *X2* | *df* | *P value* |
| --- | --- | --- | --- | --- | --- |
| Fixed Effects |  | 1148.3 | 25.59 | 1 | < .001 |
| Conflict variables | *Levels of factor* | *β* | *S.E* | *Z* | *P value* |
| ***Bystander proximity |  | -0.652 | 0.135 | -4.835 | <0 .001 |
| Redirection |  | 0.365 | 0.211 | 1.723 | 0.084 |
| **Context | Non-feed vs feed | 0.645 | 0.187 | 3.448 | <0.001 |
| Social variables |  |  |  |  |  |
| *** Vic-bystander affiliation |  | 0.523 | 0.122 | 4.255 | <0.001 |
| *Bystander age | Juvenile vs adult | 0.528 | 0.283 | 2.217 | 0.027 |
|  | ***Adoles vs adult | 0.737 | 0.215 | 3.483 | <0.001 |
| *Victim sex | Male vs female | 0.575 | 0.248 | 2.319 | 0.020 |
| **Victim-bystander kinship |  | 1.136 | 0.438 | 2.596 | 0.009 |
| Random Factors | Variance | SD |  |  |  |
| Case number | 0.144 | 0.379 |  |  |  |
| Victim identity | 0.151 | 0.388 |  |  |  |
| Bystander identity | 1.224 e-10 | 1.106 e-10 |  |  |  |
| Aggressor identity + Group | 0.000 | 0.000 |  |  |  |

Asterisks represent significance values: *** = P < .001; ** = P <.01, * = P <.05
