# Supplementary material for: Bonobos Respond to Distress in Others: Consolation across the Age Spectrum
Source: PLoS One. 2013 Jan 30;8(1):e55206. doi: 10.1371/journal.pone.0055206 (PMC3559394; doi:10.1371/journal.pone.0055206)
Supplement: Table S4 — Mean levels of self-directed behaviours in PCs with and without consolation, as compared with MCs. (DOCX) [file pone.0055206.s005.docx]

**Table S4.** Mean levels of self-directed behaviours in PCs with and without consolation, as compared with MCs**.** PCs containing reconciliation were excluded.

| Group | Condition | Mean | SD | Wilcoxon sign ranks test | Z |  |
| --- | --- | --- | --- | --- | --- | --- |
|  | Self-scratch rate (min) |  |  |  |  |  |
| 1 | PC consolation | 0.180 | 0.160 | PC_consolation * PC no consolation | -2.053 | 0.041 |
|  | PC no consolation | 0.389 | 0.561 | PC consolation * MC | -3.071 | 0.002 |
|  | MC | 0.377 | 0.225 |  |  |  |
| 2 | PC consolation | 0.216 | 0.201 | PC_consolation * PC no consolation | 0.00 | >0.05 |
|  | PC no consolation | 0.438 | 0.376 | PC consolation * MC | -2.366 | 0.018 |
|  | MC | 0.366 | 0.228 |  |  |  |
|  | Self-groom duration |  |  |  |  |  |
| 1 | PC consolation | 2.096 | 2.70 | PC_consolation * PC no consolation | -0.345 | >0.05 |
|  | PC no consolation | 2.422 | 3.663 | PC consolation * MC | -1.989 | 0.047 |
|  | MC | 3.880 | 3.479 |  |  |  |
| 2 | PC consolation | 1.730 | 2.690 | PC_consolation * PC no consolation | -0.944 | >0.05 |
|  | PC no consolation | 3.906 | 3.849 | PC consolation * MC | -0.314 | >0.05 |
|  | MC | 3.869 | 9.199 |  |  |  |
