# Supplementary material for: Bonobos Respond to Distress in Others: Consolation across the Age Spectrum
Source: PLoS One. 2013 Jan 30;8(1):e55206. doi: 10.1371/journal.pone.0055206 (PMC3559394; doi:10.1371/journal.pone.0055206)
Supplement: Table S5 — Percentage of consolatory contacts offered by mother-reared juveniles to their mothers. (DOCX) [file pone.0055206.s006.docx]

**Table S5.** Fixed and random factors entered into the GLMM analyses for occurrence of reconciliation. Factors included in the best fitting model are indicated with an asterisk.

| Fixed factors | Type |
| --- | --- |
| *Conflict variables* |  |
| ** Context* | Binomial-feed/non-feed |
| ** Redirection by victim* | Binomial-yes/no |
| ** Reconciliation* | Binomial-yes/no |
| *Support* | Binomial-yes/no |
| *Conflict Intensity* | Ordinal (1-6) |
| **Distance of bystander to conflict* | Ordinal (1-3; 1= <5m, 2=5-10m, 3= >10m) |
| *Social & relationship variables* |  |
| *Victim-bystander affiliation | Continuous, (log-transform) |
| Victim-aggressor affiliation | Continuous, (log-transform) |
| *Age victim/aggressor/*bystander | Nominal- adult/adolescent/juvenile |
| * Sex victim/aggressor/bystander | Nominal- female/male |
| Rearing victim/aggressor/*bystander | Nominal- orphan/mother-reared |
| * Kinship between victim/bystander | Binomial- yes/no |
| *Kinship between aggressor/ bystander | Binomial- yes/no |
| *Random factors* |  |
| **Group* | Nominal- Group 1 or 2 |
| ** Conflict Interaction Number* | Nominal |
| *** Victim/Bystander/Aggressor Identities | Nominal |
