# Supplementary figures and images for: Bonobos Respond to Distress in Others: Consolation across the Age Spectrum
Source: PLoS One. 2013 Jan 30;8(1):e55206. doi: 10.1371/journal.pone.0055206 (PMC3559394; doi:10.1371/journal.pone.0055206)

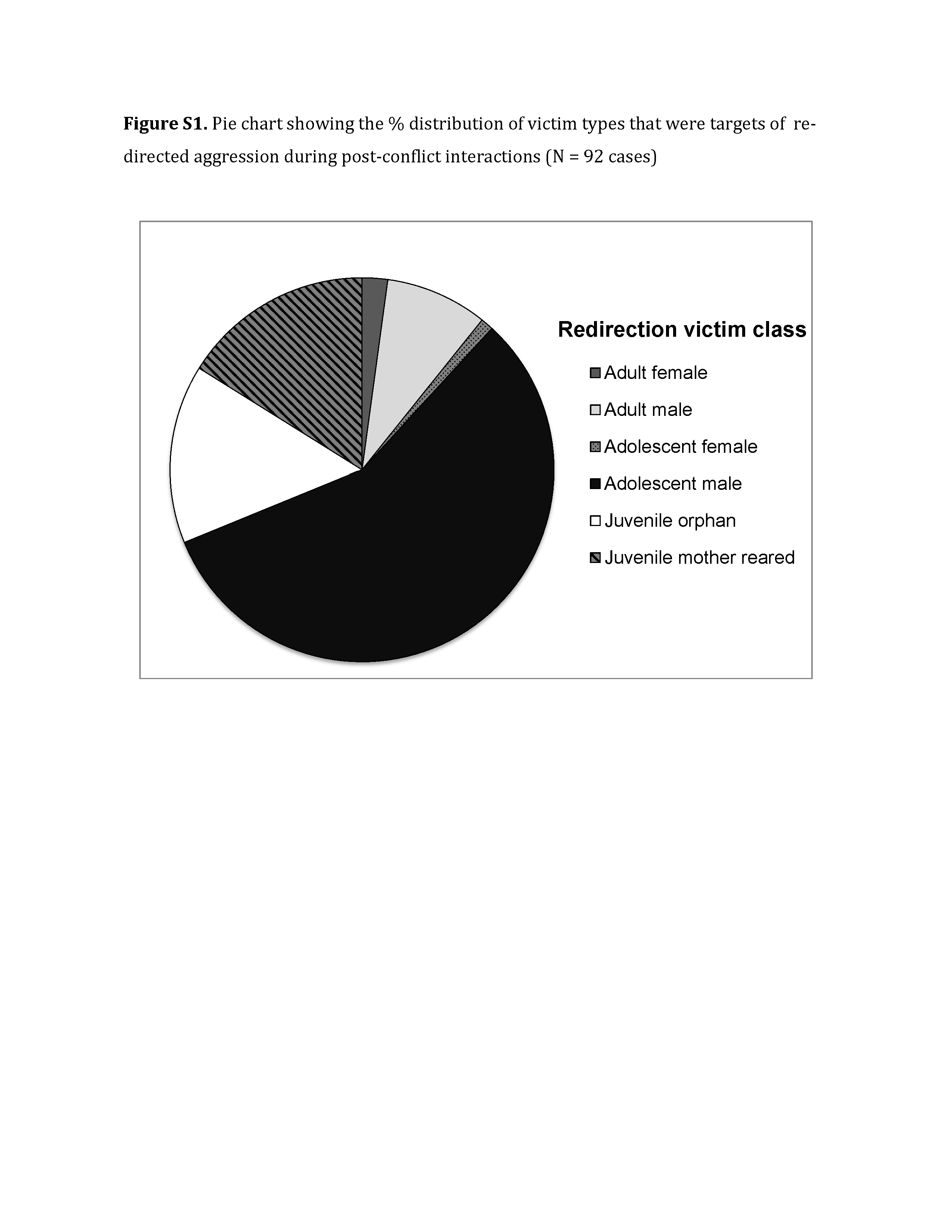

Supplement: Figure S1 — Pie chart showing the % distribution of victim types that were targets of re-directed aggression during post-conflict interactions. (TIF) [file pone.0055206.s007.tif]
